# Spatio-Temporal Profiling of *Metarhizium anisopliae*—Responsive microRNAs Involved in Modulation of *Plutella xylostella* Immunity and Development

**DOI:** 10.3390/jof7110942

**Published:** 2021-11-08

**Authors:** Junaid Zafar, Yuxin Zhang, Junlin Huang, Shoaib Freed, Rana Fartab Shoukat, Xiaoxia Xu, Fengliang Jin

**Affiliations:** 1 Laboratory of Bio-Pesticide Creation and Application of Guangdong Province, College of Plant Protection, South China Agricultural University, Guangzhou 510642, China; jz_jaam@yahoo.com (J.Z.); jzento57@gmail.com (Y.Z.); huangjunlinscau@163.com (J.H.); ranafartab@gmail.com (R.F.S.); 2 Laboratory of Insect Microbiology and Biotechnology, Department of Entomology, Faculty of Agricultural Sciences and Technology, Bahauddin Zakariya University, Multan 66000, Pakistan; sfareed@bzu.edu.pk

**Keywords:** biocontrol, midgut, small RNA, gene silencing, diamond back moth

## Abstract

*Metarhizium anisopliae*, a ubiquitous pathogenic fungus, regulates a wide array of the insect pest population. The fungus has been employed to control *Plutella xylostella*, an insecticide-resistant destructive lepidopteran pest, which causes substantial economic losses in crops worldwide. Integration of modern gene-silencing technologies in pest control strategies has become more crucial to counter pesticide-resistant insects. MicroRNAs (miRNA) play essential roles in the various biological process via post-transcriptional gene regulation. In the present study, RNA-seq analysis of control (CK36h, CK72h) and fungal-infected (T36h, T72h) midguts was performed to reveal underlying molecular mechanisms occurring in larval midgut at different time courses. We aimed at exploring *M. anisopliae*-responsive miRNAs and their target genes involved in development and immunity. After data filtration, a combined set of 170 miRNAs were identified from all libraries. Interestingly, miR-281, miR-263, miR-1, miR-6094 and miR-8 were listed among the most abundantly expressed conserved miRNAs. Furthermore, we experimentally studied the role of differentially expressed miR-11912-5p in regulating corresponding target trypsin-like serine proteinase (*Px_TLSP*). The luciferase assay (in vitro) revealed that miRNA-11912-5p significantly downregulated its target gene, suggesting it might play a crucial role in defense mechanism of *P. xylostella* against *M.+ anisopliae* infection. We used synthetic miRNA mimic/inhibitor (in vivo), to overexpress/silence miRNA, which showed harmful effects on larval duration, survival and adult fecundity. Additionally, fungal application in the presence of mimics revealed enhanced sensitivity of *P. xylostella* to infection. Our finding provides an insight into the relatively obscure molecular mechanisms involved in insect midgut during the fungal infection.

## 1. Introduction

*Plutella xylostella* (L.) (Lepidoptera: Plutellidae) is a major pest of cruciferous crops distributed throughout the world, causing severe economic damages. Imprudent use of chemical insecticides has led to various complications, such as environmental pollution and pesticide resistance [1,2]. Over the past few decades, the pest has developed resistance against various families of synthetic insecticides [3]. Arthropod Pesticide Resistance Database (APRD) ranked *P. xylostella* among the top 20 most resistant species [4]. *P. xylostella* was also the first insect species to have developed resistance against toxins from *Bacillus thuringiensis* [5]. Therefore, to combat resistance development, it is essential to probe for alternative control measures.

Scientists have explored entomopathogenic fungi due to their ability to control susceptible, resistant, and multi-resistant arthropod populations [6,7]. These microbial insecticides attach to the host cuticle, germinate and penetrate hemolymph, leading to fungal growth inside the host, eventually producing and dispersing secondary infectious conidia [8]. Entomopathogenic fungi, *Beauveria bassiana* and *Metarhizium anisopliae*, have shown potential against various pests, including *Aedes albopictus* [9], *Bemisia tabaci* [10], *Culex pipiens* [11] and *Spodoptera frugiperda* [12]. Insects respond to pathogenic infections by initiating various immune pathways, activating transcription factors, and gene expressional changes [13]. Our recent study found that *M. anisopliae* could effectively control the *P. xylostella* population [14]. However, a comprehensive understanding of the molecular interplay between host–pathogen is required, which will help unravel innovative methods for incorporating fungus into pest management programs.

MicroRNAs (miRNAs) comprise a large family of single-stranded small non-coding RNAs (∼22 nucleotides) that regulate the expression of genes by binding complementarily to the target sites in the mRNAs [15]. They are involved in the modulation of various biological processes, such as metabolism, immunity [16], and development [17] via post-transcriptional regulation either by obstructing mRNA translation or degrading it [18]. Suppression and overexpression of miRNAs can severely affect the normal development in insects, with potentially fatal consequences [19]. The use of synthetic miRNA mimics (overexpression) and inhibitors (suppression) has been proven prolific by targeting key genes [20]. In migratory locusts, miR-71 and miR-263 expressed in epidermis upregulate and produce molting-defective phenotypes [21]. In *Bombyx mori*, miR-14 overexpression leads to delayed larval development and produces smaller-sized larvae and pupa [22].

Entomopathogenic fungi have been extensively studied for host–pathogen interactions occurring at insect cuticle and hemolymph regulated by miRNAs [23,24]. However, the evidence regarding the immune and developmental challenges caused by entomopathogenic fungi in insect midgut, a key organ for various physiological processes such as development [25], immunity [26], reproduction [27], and stress response [28], is scarce. Even though researchers have investigated the possibility of oral infection in terrestrial arthropods, but the evidence suggests that even if ingested, *Metarhizium* spp. fails to germinate in the host gut. It could be attributed to a hostile environment (unfavorable digestive enzymes and pH) present in the insect gut. However, evidence suggests that *B. bassiana* can infect through the gut, chiefly due to its toxic genes (*Vip3A* and Cry-like delta endotoxins) shared with bacterial pathogens [29,30].

Despite penetration through the cuticle, fungal pathogens can also manipulate gut activities. For instance, entomopathogenic fungi modulate gut microbes and accelerate mortality [31]. Histological evidence suggests that entomopathogenic fungi, *B. bassiana*, destroy midgut epithelium after penetration through the cuticle, displacing columnar layer towards gut lumen with an increasing number of regenerative cells beneath it [32]. In *Spodoptera littoralis* larvae, when infected with three species of entomopathogenic fungi, i.e., *Paecilomyces fumosoroseus*, *B. bassiana*, and *M. anisopliae* lead to exfoliation of the midgut epithelium and disturbance of peritrophic membrane [33]. Fungal hyphae of *B. bassiana* colonized in the midgut musculature of termite, *Odontotermes obesus* followed by penetration into the gut lumen through epithelial cells and caused columnar cells to slough off, becoming disorganized, granular and hypertrophied [34]. Similarly, in *B. mori*, entomopathogenic fungi severely damaged the midgut tissues resulting in hyphal growth and ultimately death [35], signifying the importance of midgut during fungal infection. We theorized that if entomopathogenic fungi can cause dysbiosis, blockage and destruction of midgut epithelium, it is plausible that it could trigger the miRNA–mRNAs involved in the gut immunity and development.

The objective of the current study is to investigate the spatiotemporal host–pathogen interaction occurring within midgut after infection with *M. anisopliae* using next-generation RNA sequencing. Additionally, bioinformatics tools identified differentially expressed miRNAs and their potential target genes. Furthermore, we used different molecular approaches to study the miRNA–mRNA interactions. Finally, the suppression and overexpression of miRNA and its consequences on various biological parameters were explored.

## 2. Materials and Methods

### 2.1. Fungal Infection and Small RNA Sequencing

Entomopathogenic fungi, *M. anisopliae*, was used to infect the 3rd instar larvae of *P. xylostella*, while aqueous 0.05% Tween-80 (Sigma-P1754) was taken as control as described in our previous studies [14]. Midgut tissues from control (CK36h and CK72h) and infected (T36h and T72h) groups were dissected in phosphate buffer saline (PBS) using a sterilized dissection kit and snap-frozen in liquid nitrogen. Trizol Total RNA isolation Kit (Takara, Japan) was used to extract total RNA following the manufacturer’s instructions. The concentration and integrity of RNA were determined by Nanodrop (Bio-Rad, Hercules, CA, USA) and Agilent 2100 Bioanalyzer (Agilent, Palo Alto, CA, USA), respectively. Briefly, RNAs were first ligated with a 3′ adapter followed by size fraction and ligation to 5′ adapter. Then, the small RNA libraries were constructed and sequenced using Illumina HiSeq^TM^ 2500 by Gene Denovo Biotechnology Co. (Guangzhou, China).

### 2.2. Bioinformatics Analysis of Small RNA Sequences

To obtain clean data, raw reads were filtered to remove low-quality transcripts, removing reads containing 3′ and 5′ adapter and transcripts shorter than 18 nucleotides. High-quality reads were then mapped to the *P. xylostella* genome (GCA_000330985.1), and clean tags were aligned to the Rfam database (11.0) to identify and remove rRNA, scRNA, snoRNA, snRNA, and tRNA. Finally, the miRBase (Version 21) database was used to identify known miRNAs, while unannotated clean sequences were used to predict novel transcripts using Mireap_v 0.2 [36].

Three software, RNAhybrid, miRanda, and TargetScan were used to analyze and predict potential targets of *M. anisopliae*-responsive miRNAs. The intersection of these software was considered more credible and chosen as predicted miRNA target genes. Additionally, the Gene Ontology (GO) database and Kyoto Encyclopedia of Gene & Genomes (KEGG) pathway enrichment analyses of all the predicted genes were performed for functional annotation. Differential expression analysis was performed by transforming the read counts into transcript per million (TPM) values. The corrected *p* ≤ 0.05 was set as the threshold to determine significant enrichment of the gene sets.

### 2.3. RT-qPCR Validation

To validate RNA-sequencing results, real-time quantitative PCR (RT-qPCR) was used. Subsequently, miRNAs and their targets were randomly selected, and RT-qPCR was performed using a Bio-Rad iQ2 optical system (Bio-Rad, Hercules, CA, USA) and SsoFast EvaGreen Supermix (Bio-Rad, Hercules, CA, USA) while U6/RPS13 were used as an internal control. The reaction protocol is as follows: 95 °C for 30 s, 40 cycles of 95 °C for 5 s, and 55 °C for 10 s, and a dissociation curve was generated from 65 to 95 °C to confirm the purity [37]. In addition, among the differentially expressed miRNAs, miR-11912-5p and its target gene trypsin-like-serine proteinase (*Px_TLSP*) were expressed during both time points and was selected for further functional studies. Data analysis was performed using the 2^−ΔΔCT^ method [38].

### 2.4. In Vitro Luciferase Validation

The HEK293T cells used in the current study were cultured in Dulbecco’s Modified Eagle Medium (DMEM) (Gibco, Grand Island, NY, USA) and supplemented with 10% fetal bovine serum. Cells were maintained at 37 °C with 5% CO_2_. The binding site of *Px*_*TLSP* (Gene ID: 105383574) was amplified and cloned into psiCHECK2 vector (Promega, Madison, WI, USA) containing the restriction enzymes sites *Not*I and *Xho*I. A mimic of miR-11912-5p was designed and synthesized (GenePharma, Shanghai, China).

The cells were co-transfected with 800 ng of the reporter vector and miRNA mimic at the ratio of 1:3 using Lipofectamine™ 2000 reagent (Invitrogen, Waltham, MA, USA) following the manufacturer’s guidelines. Cells were transfected for 48 h and harvested for dual luciferase assay. Renilla luciferase and Firefly activities were measured using the Dual-GLO^®^ Luciferase Assay System (Promega, USA) with a luminometer (Promega, USA). Results are expressed as the ratio Renilla/firefly luciferase activity (Mean ± SEM) at *p* < 0.01. Primer sequences and the mimics/inhibitors used in this study are given (Table S7).

### 2.5. Overexpression and Inhibition Treatment of miRNA In Vivo

The synthetic miRNA mimics and inhibitors were used to perform the functional studies of miRNA in vivo. Briefly, 1 g artificial diet prepared in RNAse-free water was enriched with 20 µL miR-11912-5p mimics (20 µM), and 10 µL inhibitor (20 µM) [39]. Susceptible 3rd instar larvae (*n* = 60) were fed on the treated and control diet for 24 h. The successful delivery of mimics/inhibitors was verified using RT-qPCR. The digestive activity of *Px_TLSP* in the mimic treated larval stage was measured using Nα-Benzoyl-DL-Arg-p-nitroanilide (BAPNA, Sigma). Next, 4 mM BAPNA was prepared in 100 mM Tris, pH 8.0, which contained 20 mM CaCl_2_ and was used as the substrate solution [40]. The gut was homogenized in PBS and incubated at 37 °C. The experiments were performed in microtiter plates with a final volume of 100 µL containing 10 µL gut content and 40 µL substrate. The absorption was measured at 405 nm using a microplate reader (Bio-Rad). Furthermore, larval duration and mortality were calculated among all the groups. The adults, upon emergence, were paired (1 pair/cage) for egg-laying, and average fecundity was calculated on a daily basis.

### 2.6. Fungal Bioassay

To investigate the role of miR-11912-5p in *P. xylostella* larval susceptibility to entomopathogenic fungi, *M. anisopliae* was used as an infection. Third instar larvae were fed on a diet mixed with mimic, control mimic, and control, and later infected with the fungal solution. Mortality data were recorded every 24 h. Larvae with no movement were considered dead. Cadavers were placed in humid chambers to observe conidial growth.

## 3. Results

### 3.1. sRNA Sequencing Analysis

Small RNA libraries were constructed to identify miRNAs in *M. anisopliae*-infected *P. xylostella* larval midgut using next-generation Illumina RNA sequencing. Totally, 16,450,812 (CK36h) 17,025,780 (T36h), 16,294,807, (CK72h), and 18,257,426 (T72h) high-quality reads were obtained. After quality check by filtering the sequences < 18 nt, adapters, and low-quality reads, a total of 10,693,027 (CK36h), 10,814,199 (T36h), 12,711,662 (CK72h), and 13,740,439 (T72h) were obtained for further analysis (Table S1). The clean reads were then mapped to the *P. xylostella* genome (GCA_000330985.1) resulting in 6,491,737 (60.71%) 7,288,770 (67.40%), 9,135,560 (71.86%), 9,782,371 (72.56%) matched reads in CK36h, T36h, CK72h, and T72h, respectively (Table S2).

Subsequently, the clean reads were categorized into miRNA, rRNA, snRNA, snoRNA, rRNA, tRNA, and unannotated ensuing priority rule of rRNA, etc.; GenBank > Rfam) > known miRNA > repeat > exon > intron. All the clean reads were divided into different categories including miRNA, rRNA, snRNA, snoRNA, tRNA, and unannotated (unann) following priority rule of rRNA etc.; (GenBank > Rfam) > known miRNA > repeat > exon > intron [41]. The overall sRNA class composition of each library is presented (Figures S1 and S2).

### 3.2. Identification of M. anisopliae Responsive Known and Novel miRNAs

**Figure 1:**
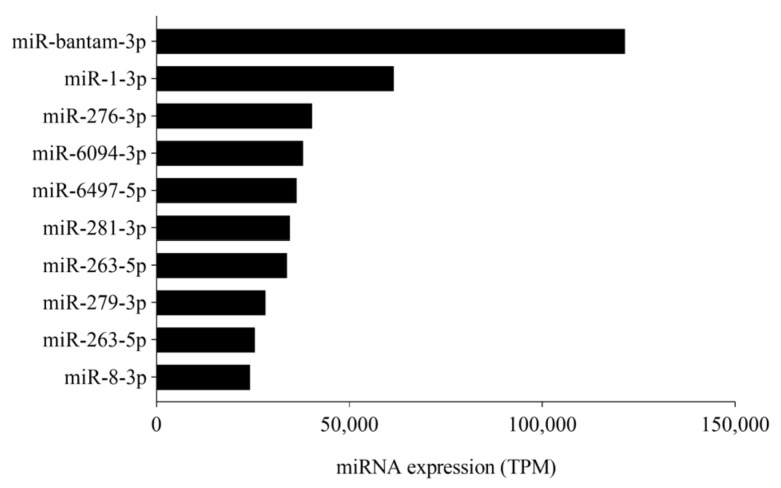
Top 10 abundantly expressed miRNAs in *P. xylostella* after *M. anisopliae* infection over the time course. Average expression values of time courses (36 h and 72 h) are presented. TPM denotes transcript per million.

We filtered and compared the miRNA sequences from previously reported [42] and identified known miRNAs; after filtering those with read count < 10 in all libraries, 127 known miRNAs were found (Table S3). Notably, the abundantly expressed miRNAs, included miR-bantam-3p, miR-281-3p, miR-1-3p, miR-8-3p, and miR-6094-3p, in response to *M. anisopliae* infection. The top 10 abundantly expressed miRNAs are presented in Figure 1. The remaining unmatched sequences were used to predict novel miRNAs using Mireap_v 0.2 following standard criteria, which resulted in the identification of 43 novel miRNAs (Table S4).

### 3.3. Identification of Differentially Expressed miRNAs and Target Predictions

Differential expression analysis was carried out to determine variations in miRNA levels followed by infection from *M. anisopliae*. After normalization of read counts by transforming them into transcript per million (TPM) values, we found that 20 (T36h) and 15 (T72h) known miRNAs were identified as differentially expressed, compared to control (Table S5). Similarly, 12 (T36h) and 14 (T72h) novel miRNAs were also identified as differentially expressed (Table S6). Overall, most of the differentially expressed miRNAs were upregulated, followed by fungal infection and gradually downregulating as time progressed.

### 3.4. RT-qPCR Validation of miRNAs and Their Targets

**Figure 2:**
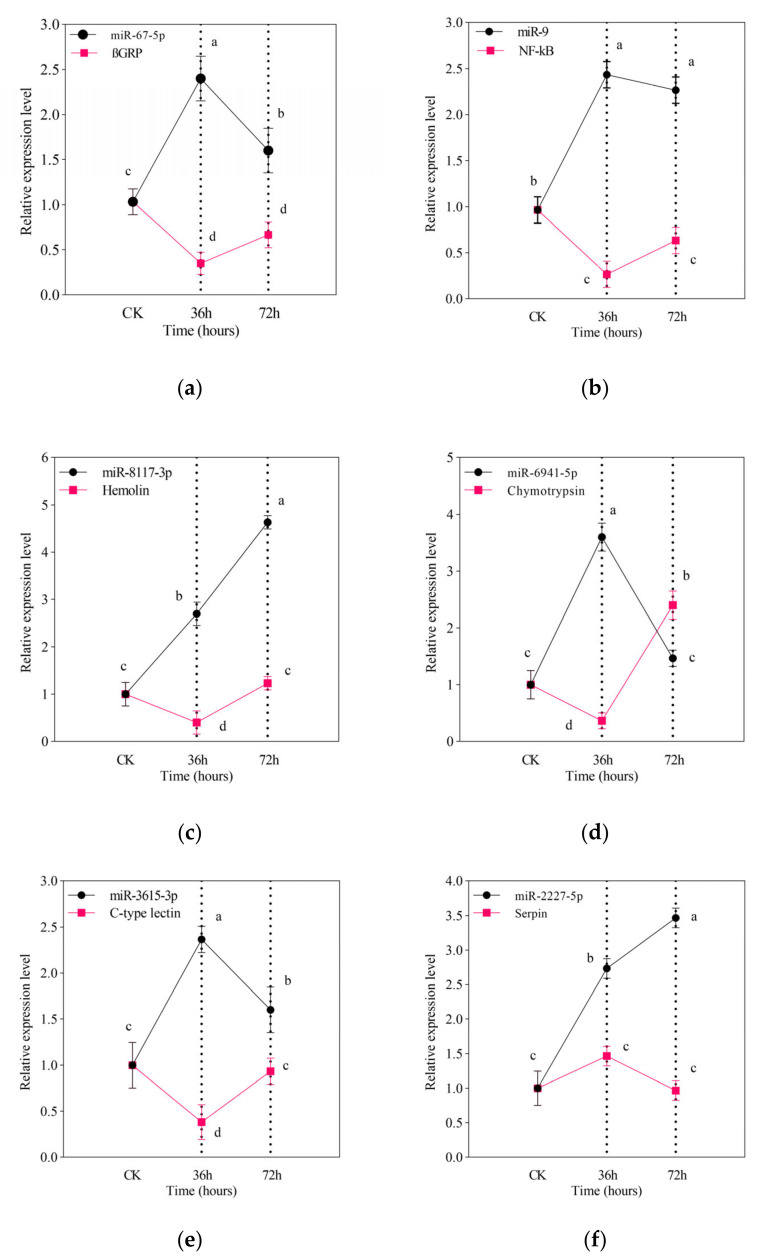
RT-qPCR validation of known miRNAs and their targets mRNAs. (**a**) miR-67-5p target βGRP; (**b**) miR-9 target NF-κB; (**c**) miR-8117-3p target Hemolin; (**d**) miR-6941-5p target Chymotrypsin; (**e**) miR-3615-5p target C-type lectin; (**f**) miR-2227-3p target Serpin followed by *M. anisopliae* infection at different course (36 and 72 h). Relative fold change of mRNA and miRNAs were normalized using RPS13 and U6, respectively, as an internal control. Error bars show 95% confidence intervals (CI). Letters indicate significant differences at *p* < 0.001.

To validate the identified miRNAs and their respective targets, RT-qPCR was performed (Figure 2). We focused mainly on immunity and development-related miRNAs and their target genes from differentially expressed transcripts commonly predicted by bioinformatic tools. Negative correlation was observed between miRNAs and the target genes among the control and the treatment groups. The expression level of βGRP (β-1,3-Glucan recognition protein), a gene involved in recognition of fungal pathogen, was significantly downregulated by miR-67-5p at the initial stage of infection (36 h) with a gradual increase at 72 h. (Figure 2a). The expression level of NF-κB (nuclear factor kappa-light-chain-enhancer of activated B cells) responsible for downstream stimulation of specific immune genes and triggering of antimicrobial peptides was continually downregulated by miR-9 (Figure 2b). Similarly, the expression level of Hemolin, a gene involved in the pathogen-induced immune responses, was significantly downregulated by miR-8117-3p at 36 and 72 h post infection compared to control (Figure 2c). Chymotrypsin, a major digestive protease in larval midgut, was negatively affected by abundantly expressed miR-6941-5p (Figure 2d). However, the results showed a slight discrepancy in the RNA sequencing analysis, primarily due to the differences between the two techniques.

### 3.5. Prediction and Annotation of miRNA Targets and Functional Classification

**Figure 3:**
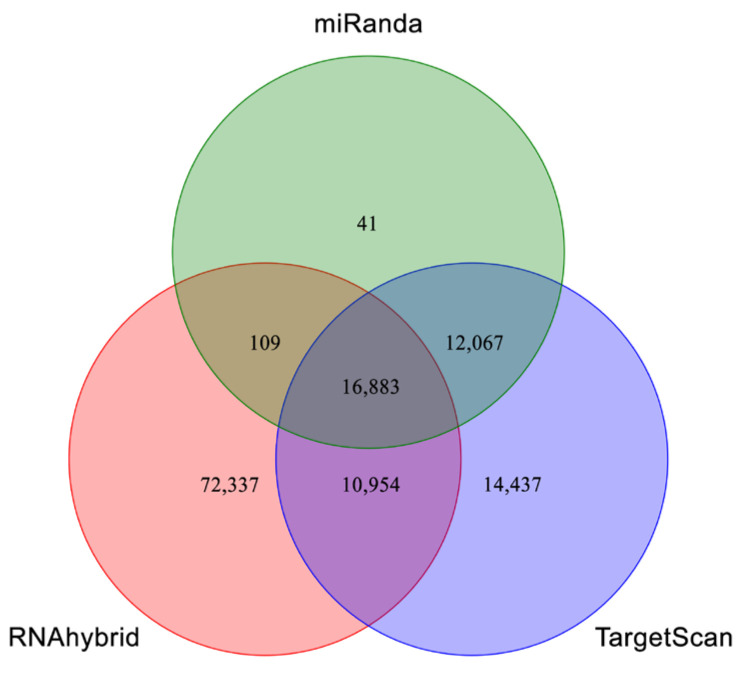
Potential target genes of miRNAs identified in *P. xylostella* followed by *M. anisopliae* infection. Venn diagram displays the number of miRNA target sites identified by each software (miRanda, TargetScan, and RNAhybrid).

We used three software (TargetScan, miRanda, and RNAhybrid) to predict the potential miRNA targets. The results showed that 16,883 shared spots as target predictions of 170 miRNAs (Figure 3). Overall, 28,950 common spots were identified between miRanda and TargetScan, 27,837 between RNAhybrid and TargetScan, and 16,992 between miRanda and RNAhybrid. GO enrichment analysis was carried out to understand the functions of target genes in biological processes, cellular components, and molecular functions (Figures S3 and S4). Furthermore, KEGG enrichment pathway analysis was used to categorize *M. anisopliae*-responsive target genes of miRNAs; the top 20 enriched categories included lysosomes, transport, various signaling pathways, and more (Figures S5 and S6).

Additionally, we compared differentially expressed genes from current studies with our previous libraries where *P. xylostella* was infected with bacterial and fungal pathogens [43,44,45] to filter out immune-related target genes. Interestingly, we found that miR-11912-5p was among the differentially expressed miRNAs in *P. xylostella* larvae during *M. anisopliae* infection, which resulted in the downregulation of its target gene trypsin-like serine proteinase (*Px_TLSP*). Trypsin is a major enzyme present in insect midgut known to perform a key role in digestion, development, and survival [46], indicating that it might play a crucial role in the defense mechanism.

### 3.6. In Vitro Luciferase Validation

**Figure 4:**
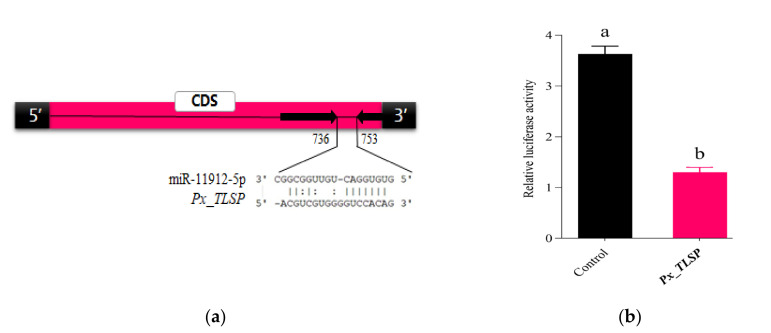
Prediction and validation of miR-11912-5p and its target site in vitro. (**a**) Predicted target site of miR-11912-5p in *Px_TLSP* by TargetScan, RNAhybrid and miRanda; (**b**) validation of miR-11912-5p-*Px_TLSP* direct sequence interaction by dual luciferase assays using HEK293T cells 48 h after co-transfection with the psi-CHECK2 construct presented as Mean ± SEM of three biological replicates. Letters indicate significant differences *p* < 0.01.

The bioinformatics tools (TargetScan, RNAhybrid, and miRanda) predicted the *Px_TLSP* as a potential target site of miR-11912-5p (Figure 4a). To experimentally verify the interaction, synthetic miR-11912-5p mimic were co-transfected with HEK293T cells. We observed significantly less luciferase activity 48 h post transfection compared to that in control (Figure 4b).

### 3.7. Overexpression and Inhibition Treatment of miRNA In Vivo

**Figure 5:**
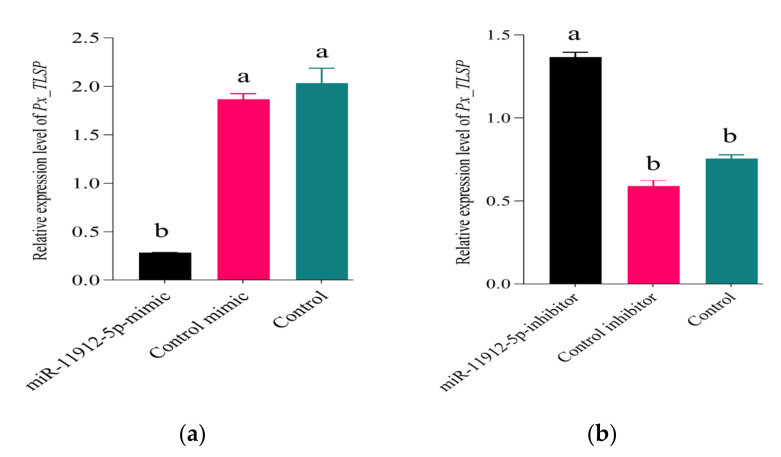
RT-qPCR expression levels of *Px_TLSP* in *P. xylostella* larvae fed on (**a**) miR-11912-5p-mimic, control mimic, control, and (**b**) miR-11912-5p-inhibitor, control inhibitor, and control. Error bars show 95% confidence intervals (CI). Letters indicate significant differences at *p* < 0.001.

To determine the effects of miR-11912-5p on its target gene in vivo, we detected the expression levels of *Px_TLSP* after miRNA mimic and inhibitor application. We quantified the expression of *Px_TLSP* in larvae fed on miR-11912-5p mimic/inhibitor (Figure 5). The relative expression levels of the *Px_TLSP* gene showed a significant reduction in mimic-fed larvae (Figure 5a), while contrastive expressions were observed in the group fed on inhibitors (Figure 5b), implying successful delivery of mimic/inhibitors in vivo.

**Figure 6:**
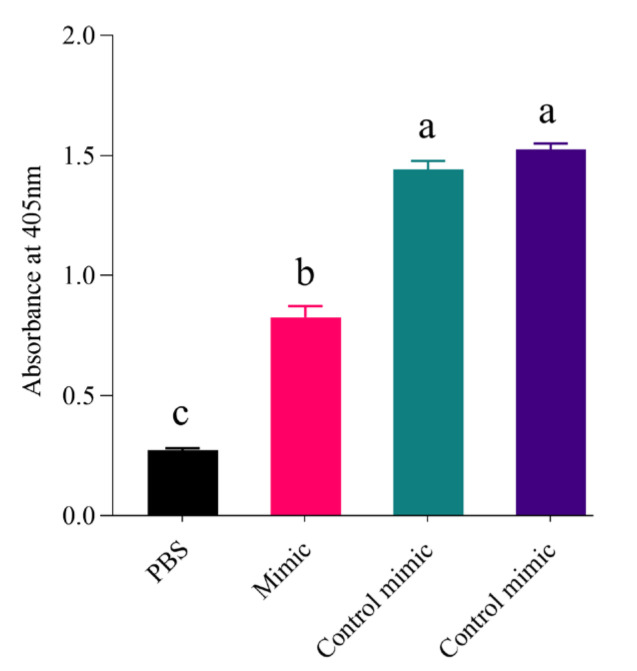
Trypsin enzyme activity in gut extracts from larvae fed on miR-11912-5p-mimic, control mimic, control, and PBS (no gut content). Three technical and two biological replicates per treatment were analyzed. Letters indicate significant differences at *p* < 0.001.

Additionally, we performed a trypsin enzyme assay to check if the decline of *Px_TLSP* in the presence of miR-11912-5p mimics parallels with low trypsin activity in the gut of *P. xylostella*. The results indicated significantly less tryptic activity in the gut extracts from larvae fed on mimic than control (Figure 6).

### 3.8. Impact on Development, Fecundity, and Survival

**Figure 7:**
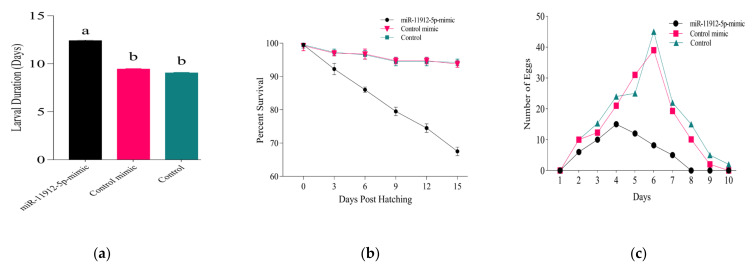
Impact of mimic administration on *P. xylostella* larval development, survival and fecundity. (**a**) average larval duration; (**b**) survival percentage of *P. xylostella* larvae fed on miR-11912-5p-mimc, control mimic and control; (**c**) average adult fecundity from start of the egg laying. Error bars represent standard deviations of mean values from independent replicates. Letters indicate significant differences at *p* < 0.05.

Trypsin has been involved in the regulation of development and digestion [47]; we theorized that the application of synthetic miR-11912-5p mimic and inhibitor could potentially impact the larval development. Treatment of *P. xylostella* larvae with miR-11912-5p mimic resulted in detrimental effects on larval development, fecundity, and survival (Figure 7). The increased larval duration (12 d) was observed in mimic-treated larvae compared to control mimic and control (9 d) (*p <* 0.0001) (Figure 7a). The silencing of trypsin also showed drastic effects on survival of *P. xylostella*. The larvae fed on control mimic and control led to successful pupation of 93% (on avg.) larvae (Figure 7b), whereas 68% of mimic-treated larvae entered into pupation while the remaining larvae failed mid (larval-pupation) stage and eventually died. The successfully emerged adults from the mimic-treated group were paired, and adult fecundity was documented daily. We noticed a reduction of 63% (*p <* 0.0001) in fecundity among adults that emerged from mimic fed larvae compared to control (Figure 7c).

**Figure 8:**
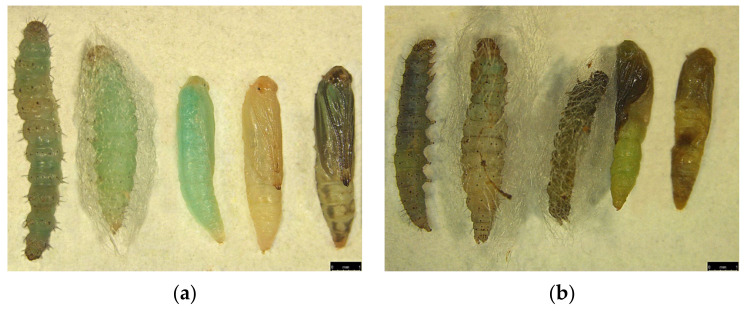
Impact of miR-11912-5p-mimic administration on *P. xylostella* larval development and pupal formation. (**a**) Larvae showing normal growth leading to successful pre-pupation and pupation in untreated population (left to right); (**b**) abnormal development during larval stage leading to failed pre-pupation and pupation in miR-11912-5p mimic-fed population (Left to Right).

Impact of silencing *Px_TLSP* using miR-11912-5p can be seen (Figure 8)**.** Abnormal larval growth and failed pupation was observed in mimic-treated group (Figure 8b) compared to control (Figure 8a) The larvae treated with miRNA inhibitor did not show any significant differences in the parameters mentioned above.

### 3.9. Fungal Susceptibility

**Figure 9:**
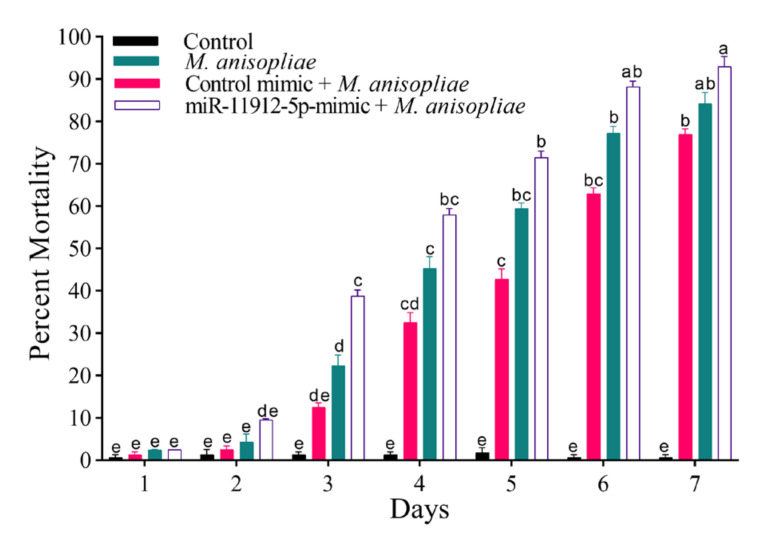
Percent mortality in *P. xylostella* followed by *M. anisopliae* infection. Mortality was recorded until seven days after every 24 h. Error bars show 95% confidence intervals (CI). Letters indicate significant differences at *p* < 0.05.

We investigated the role of mimics by enriching the larval diet and exposing the starved third instar larvae for 24 h. Larvae were then treated with *M. anisopliae* to evaluate the susceptibility. We found that larvae fed on mimic proved to be more sensitive to the fungal infection than those without (Figure 9). Maximum mean mortality (93%) was reported in the miR-11912-5p mimic, and *M. anisopliae* group, followed by 84.25% in the *M. anisopliae*-treated group, suggesting that mimic-treated larvae could potentially become more susceptible to fungal infection.

**Figure 10:**
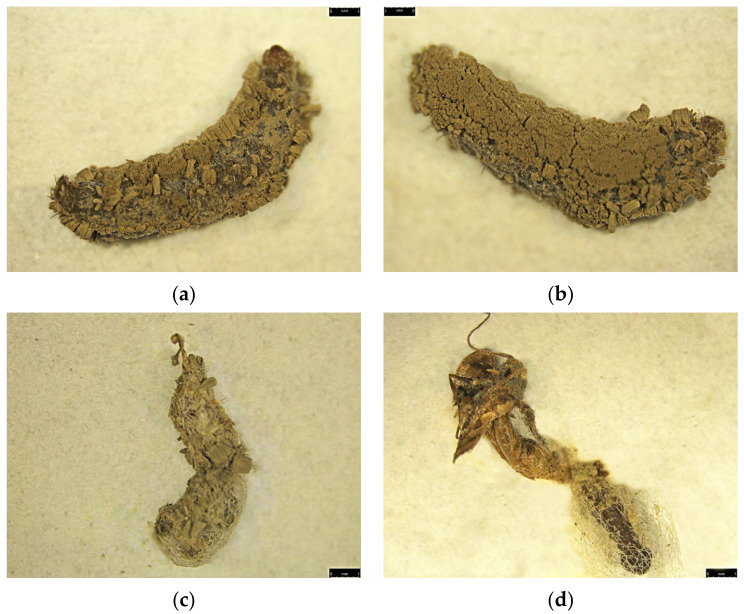
Fungal growth on various stages of *P. xylostella* infected with *M. anisopliae*. (**a**) Fungal growth over ventral region of larval body; (**b**) fungal growth over dorsal surface of larval stage; (**c**) fungal growth during pupal stage; (**d**) fungal infection resulting in failed adult emergence.

The confirmation of fungal pathogenicity was verified by placing carcasses in a humid chamber for conidial growth (Figure 10).

## 4. Discussion

The success of insects in adverse environments indicates the advanced defense mechanisms employed by these organisms, but they have often been targeted and killed by specialized pathogenic fungi. These cross-kingdom host–pathogen interactions have resulted in sustainable populations of both species. Next-generation RNA sequencing and bioinformatic tools have enabled us to unravel host–pathogen interactions in individual tissues. Entomopathogenic fungi have been frequently studied for their interaction with host integument in neutralizing their defenses, but the information regarding their impact on the host midgut is scarce. Histological and ultrastructural studies suggest that entomopathogenic fungi, *B. bassiana*, after penetration through cuticle in *C. pipiens*, significantly destroys the midgut [48]. Midgut tissues of fungi-infected *Galleria mellonella* larvae showed progressive destruction of the columnar cell after cuticular penetration [32]. Damaged crypts of the midgut and vegetative growth of entomopathogenic fungi in the digestive canal can cause mechanical blockage and is one of the reasons for the insect death after infection [49,50].

In insects, miRNAs have been involved in various biological processes such as development, immunity, reproduction, and host–pathogen interactions [51]. RNAseq analysis revealed 170 miRNAs (127 known and 43 novel) from *M. anisopliae*-infected *P. xylostella*. Bantam was the most abundantly expressed miRNA, known to perform various roles in insects such as cell proliferation, apoptosis inhibition, modulating the ecdysone signaling pathway, and immunity [52,53], implying its importance in *P. xylostella*.

The top 10 abundantly expressed miRNAs in our studies, e.g., miR-281, miR-263, miR-8, and miR-279, have been previously described among highly expressed miRNAs in *P. xylostella* [54,55]. These miRNAs are known to play critical regulatory roles in different insects [56]. For instance, miR-8 increases the susceptibility of *P. xylostella* larvae to entomopathogenic fungi by regulating the Toll pathway and negatively affecting antimicrobial peptides (AMP) [20]. Similarly, miR-281, a midgut-specific miRNA was also listed in abundantly expressed miRNAs. In *A. albopictus*, the dengue virus (DENV) promotes its replication by exploiting miR-281, suggesting that pathogen can manipulate host miRNA for its advantage [57]. Likewise, miR-14 was also found to be abundantly expressed in our studies. MiR-14, investigated from the larval midgut of lepidopteran pest *S. frugiperda*, showed high expression levels in the sixth instar, potentially regulating metamorphosis [58]. It is plausible that these miRNAs play critical modulatory roles in the midgut of *P. xylostella* larvae during fungal infection. Moreover, miR-263 and miR-279 have been detected previously from *P. xylostella* infected with destruxin, a key virulence factor of entomopathogenic fungi, *M. anisopliae* [43]. Similarly, let-7, another conserved miRNA known to play an important role in development [59] and immunity [60], was also recorded among the abundantly expressed miRNAs, inferring its critical role in *P. xylostella* immunity and development. The novel miRNAs found in our studies showed comparatively low expression levels as opposed to the conserved, possibly due to the participation of conserved miRNAs in different biological processes [61].

Fungal infections can lead to changes in the expression level of miRNAs in several insect species [62]. Several differentially expressed miRNAs were found in the current study, followed by infection from entomopathogenic fungi, *M. anisopliae*. MiR-92 expression level upregulated upon pathogen infection, and its role has been examined in *A. albopictus* and *Culex quanificatus* development [63], signifying its potential in host–pathogen interaction. Similarly, miR-317 was among differentially expressed miRNAs at 36 h, which is known to negatively modulate *Drosophila* toll immune responses and downregulate AMP productions [64], indicating its critical role in *P. xylostella* survival. Additionally, miR-8517 found in our studies have been reported previously from the pathogen-infected midgut of *P. xylostella* [65]. However, miR-2, a conserved miRNA, was listed amongst abundantly expressed miRNA, yet its expression level did not vary among treatments. Similar to our findings, miR-2 has shown high expression levels in different insect libraries including *Apis mellifera, B. mori,* and *Tribolium casteneum* [66]. The miR-2 family has been functionally studied for a critical role in *Blattella germanica* metamorphosis [67].

MiRNA mimics/inhibitors have been effectively used to establish the role of miRNAs in various insects [68,69]. In the present study, bioinformatic tools predicted that miR-11912-5p could potentially target *Px_TLSP* (trypsin-like serine proteinase), followed by infection. Proteases, especially trypsin and chymotrypsin, are predominately present in insect midgut during larval stages and play a central role in the biological process, such as digestion and development [70]. In previous studies, trypsin-like serine proteases were among differentially expressed genes in *P. xylostella* larvae followed by entomopathogenic fungi, *Isaria fumosorosea* [44] infection, suggesting their critical role. To experimentally validate the role of miR-11912-5p in the regulation of *Px_TLSP* in vitro, we performed dual luciferase assay. Our results indicated that miR-11912-5p could significantly downregulate *Px_TLSP*, suggesting its potential role in fungal infection. Similarly, miR-2b-3p could significantly downregulate the expression level of trypsin followed by pathogen infection in *P. xylostella* larvae, supporting our findings [45].

Oral administration of miR-11912-5p mimic significantly affected larval development, survival, and adult fecundity. We observed a substantial increase in larval duration, whereas 32% of larvae failed to survive and died between the intermediate (larvae–pupae) stage. Similarly, we observed reduced fecundity, as previously reported in *H. armigera* where trypsin-like serine protease (*Ha-TLP*), present in the gut, was silenced by har-miR-2002b mimics, which led to drastic effects on larval development and adult fecundity while also causing significant mortality [71]. Gene silencing technology can be used as a powerful method for controlling pests by regulating vital genes [72]. *Spodoptera exigua* larvae was adversely affected and caused significant mortality when it was orally fed with synthetic miRNA mimics (miR-9, miR-10-1a, and miR-4924), supporting our current findings [73]. Similarly, transgenic tobacco plants were developed capable of producing miR-24, which could potentially inhibit the *H. armigera* molting process with fatal consequences [74]. In rice plants, a cowpea trypsin inhibitor (CpTI) has shown significant resistance against rice borers [75]. Likewise, a trypsin inhibitor purified from *Sapindus mukorossi* (SMTI) has proven effective against *Bactrocera cucurbitae*, affecting larval growth and development [76].

Furthermore, mimic-fed *P. xylostella* larvae showed enhanced sensitivity to *M. anisopliae* infection, signifying the regulatory role of miRNA. *Meterhizium* spp. can potentially sense defense molecules produced by insects, including AMP and proteinase inhibitors and then launch a counterattack by releasing its proteinases and degrading these compounds [77]. In *P. xylostella*, miR-7a, miR-8519, and miR-189942 mimic application-increased susceptibility of insecticide resistant population [18,78]. The effects of *M. anisopliae* in midgut of migratory locust was investigated and it was reported that trypsin and chymotrypsin activity varied upon infection [79], suggesting the complex host–pathogen interaction.

## 5. Conclusions

In conclusion, we identified 127 known and 43 novel miRNAs from *M. anisopliae*-infected *P. xylostella* larval midgut. Additionally, miR-11912-5p in vitro validation revealed its significant interaction with trypsin-like serine proteinase (*Px_TLSP*) after fungal infection. We used synthetic mimic/inhibitors to overexpress and silence miRNA in vivo, which adversely affected larval duration, survival, and adult fecundity. The application of *M. anisopliae* in presence of mimic indicated that it could potentially enhance susceptibility towards infection. The study provides crucial information in establishing the modern gene-silencing technologies to counter resistant insect pest infestations. The current study also laid the foundation for further research to understand the spatiotemporal host pathogen interactions occurring in insect midgut, a vital organ for several biological processes.

## Data Availability

Not applicable.

## Supplementary Materials

The following are available online at https://www.mdpi.com/article/10.3390/jof7110942/s1: Table S1: The classification of total small RNA tags of the *Plutella xylostella*; Table S2: The mapping statistics of sRNAs from libraries of *Plutella xylostella*; Table S3: Expression profiles of *Metarhizium anisopliae*-responsive known miRNAs; Table S4: Expression profiles of *Metarhizium anisopliae*-responsive novel miRNAs; Table S5: Differential expression of *Metarhizum anisopliae*-responsive known miRNAs; Table S6: Differential expression of *Metarhizium anisopliae*-responsive novel miRNAs; Table S7: The primers used in the current study; Figure S1: Small RNA composition of control (CK36h) and treatment (T36h) in all replicates post-infection; Figure S2: Small RNA composition of control (CK72h) and treatment (T72h) in all replicates post-infection; Figure S3: Gene ontology annotation of target genes of miRNAs in *Plutella xylostella* after *Metarhizium anisopliae* infection (CK36h vs. T36h); Figure S4: Gene ontology annotation of target genes of miRNAs in *Plutella xylostella* after *Metarhizium anisopliae* infection (CK72h vs. T72h); The abscissa is the GO annotation and the ordinate left is the gene number; Figure S5: Kyoto Encyclopedia of Genes & Genomes classification of target genes in *Plutella xylostella* after *Metarhizium anisopliae* infection (CK36h vs. T36h); Figure S6: Kyoto Encyclopedia of Genes & Genomes classification of target genes in *Plutella xylostella* after *Metarhizium anisopliae* infection (CK72h vs. T72h); The abscissa is the KEGG classification and the ordinate left is the gene number.
