# Supplementary material for: Spatio-Temporal Profiling of Metarhizium anisopliae—Responsive microRNAs Involved in Modulation of Plutella xylostella Immunity and Development
Source: J Fungi (Basel). 2021 Nov 8;7(11):942. doi: 10.3390/jof7110942 (PMC8620415; doi:10.3390/jof7110942)
Supplement: Supplementary file 1 [file jof-07-00942-s001.zip › Table S7. Primers used in the study.pdf]

Table S7: Primers used in the study

Name	Primer sequences (5´- 3´)
Trypsin Psi2	*CCGCTCGAGATGAGCCTCCGTGGGGAGGGC*
	AAATGCGGCCGCGACGACATTGCTCAAAATCC
Mimic	*GUGUGGACUGUUGGCGGC*
	CGCCAACAGUCCACACUU
Inhibitor	*GC CGCC AACA GUCC ACAC*
Mimic Control	*UUCUCCGAACGUGUCACGUTT*
	ACGUGACACGUUCGGAGAATT
Inhibitor Control	*CAGUCUUUUGUGUAGUACAA*
Trypsin	*TCATCGGTGGAGAGAACGTATC*
	ACGAAGAAGGGCACAAAGC
Chymotrypsin	*TGGTGCGTCGTCCGTTAGTG*
	GCCAAAATGTACCGCCTCTA
NF-κB	*ATCAGCCATGCAGTTCCTCT*
	TGGCACAGACAGAGTTGGAG
C-type Lectin	*TGCCAGAGACACAGGAACAATCG*
	AGTGGTCGTCGAGGCTGCTG
βGRP	*CGACAGGATGATGGGGAGT*
	GGTCTGGCTTCTTGGTAGG
Serpin	*CAGGCAAGGACTCAAGTATAG*
	CTTCTACGCCATTCTTCATCA
Hemolin	*AGCTCCAGAGACTACGCCT*
	TGCACTTCAGGCTTCGGAT
RPS-13	*TCAGGCTTATTCTCGTCG*
	GCTGTGCTGGATTCGTAC

Underline = Restriction enzymes
