# Supplementary figures and images for: Spatio-Temporal Profiling of Metarhizium anisopliae—Responsive microRNAs Involved in Modulation of Plutella xylostella Immunity and Development
Source: J Fungi (Basel). 2021 Nov 8;7(11):942. doi: 10.3390/jof7110942 (PMC8620415; doi:10.3390/jof7110942)

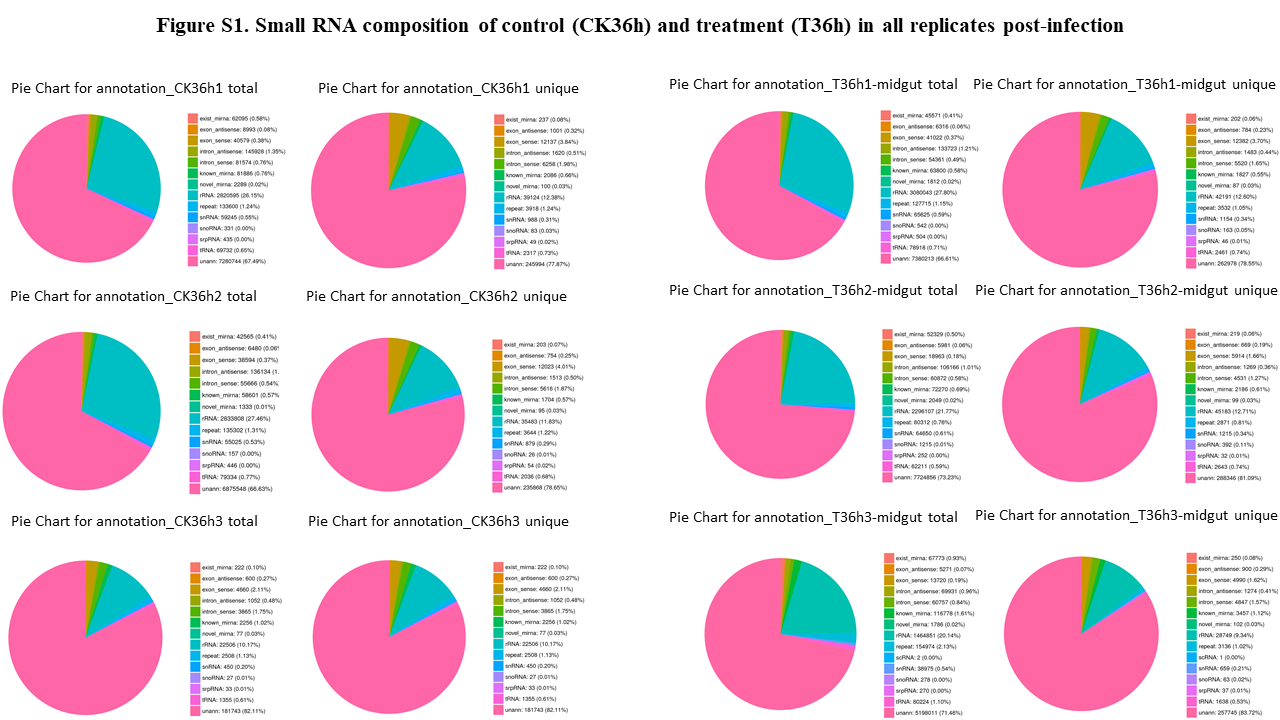

Supplement: Supplementary file 1 [file jof-07-00942-s001.zip › Figure S1 Small RNA composition of control (CK) and treatment (M) in all replicates post-infection.PNG]

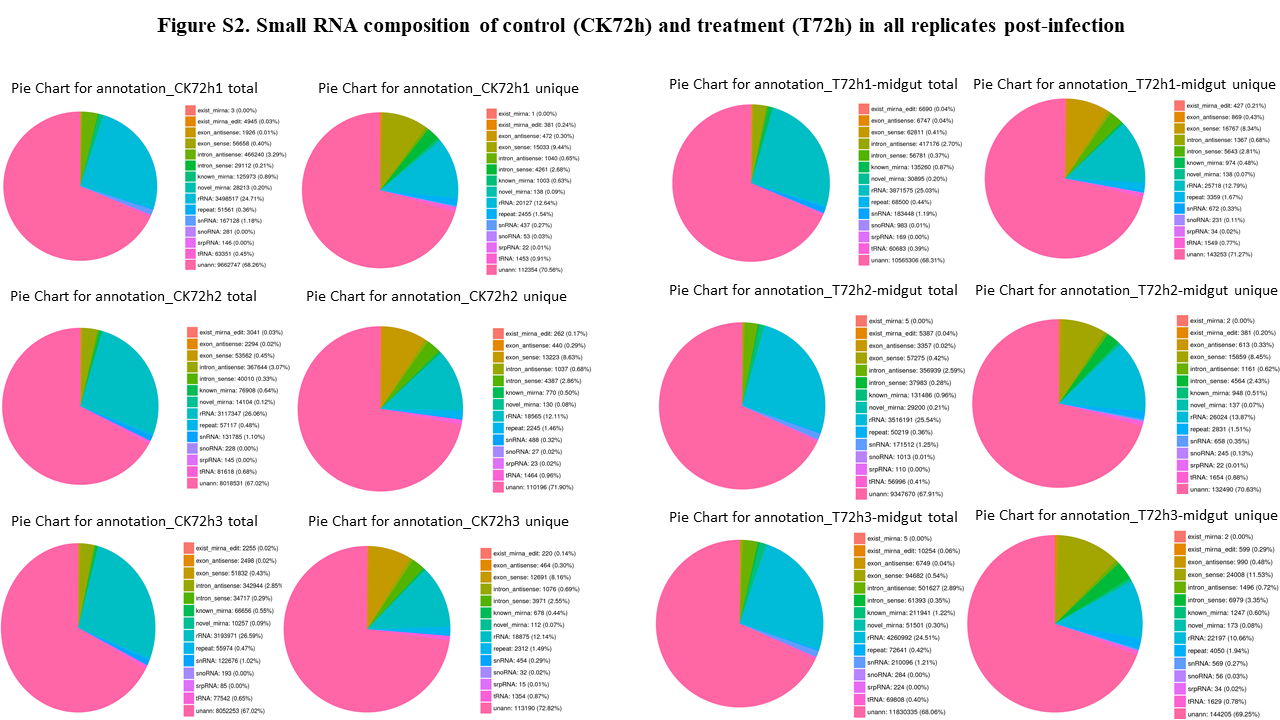

Supplement: Supplementary file 1 [file jof-07-00942-s001.zip › Figure S2 Small RNA composition of control (CK) and treatment (M) in all replicates post-infection.PNG]

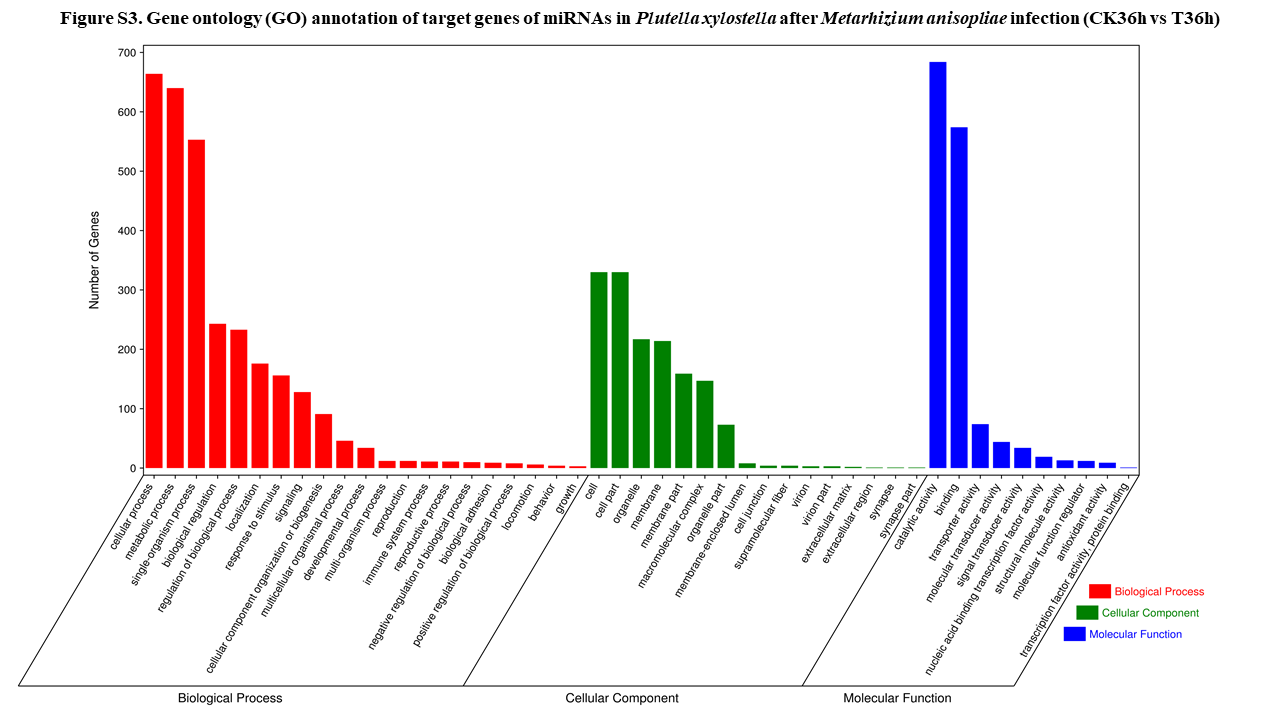

Supplement: Supplementary file 1 [file jof-07-00942-s001.zip › Figure S3 Gene ontology annotation of target genes of miRNAs in Plutella xylostella after Metarhizium anisopliae infection (CK36h vs T36h).PNG]

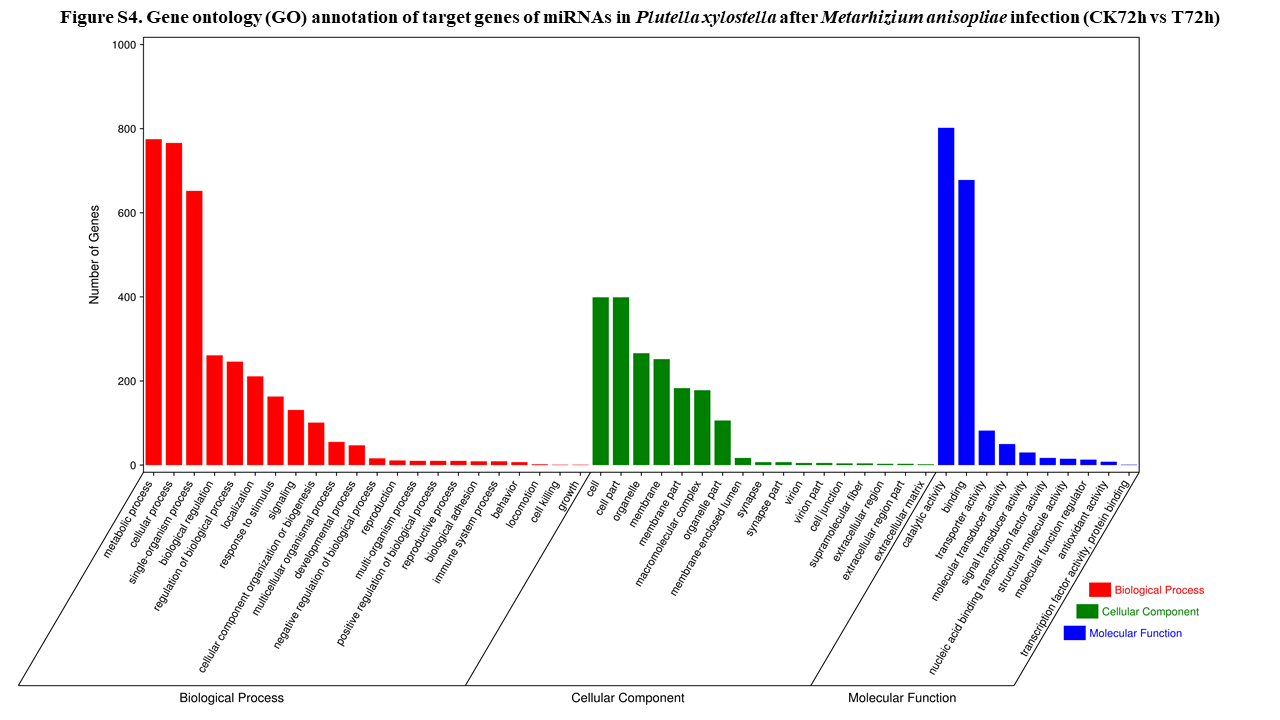

Supplement: Supplementary file 1 [file jof-07-00942-s001.zip › Figure S4 Gene ontology annotation of target genes of miRNAs in Plutella xylostella after Metarhizium anisopliae infection (CK72h vs T72h).PNG]

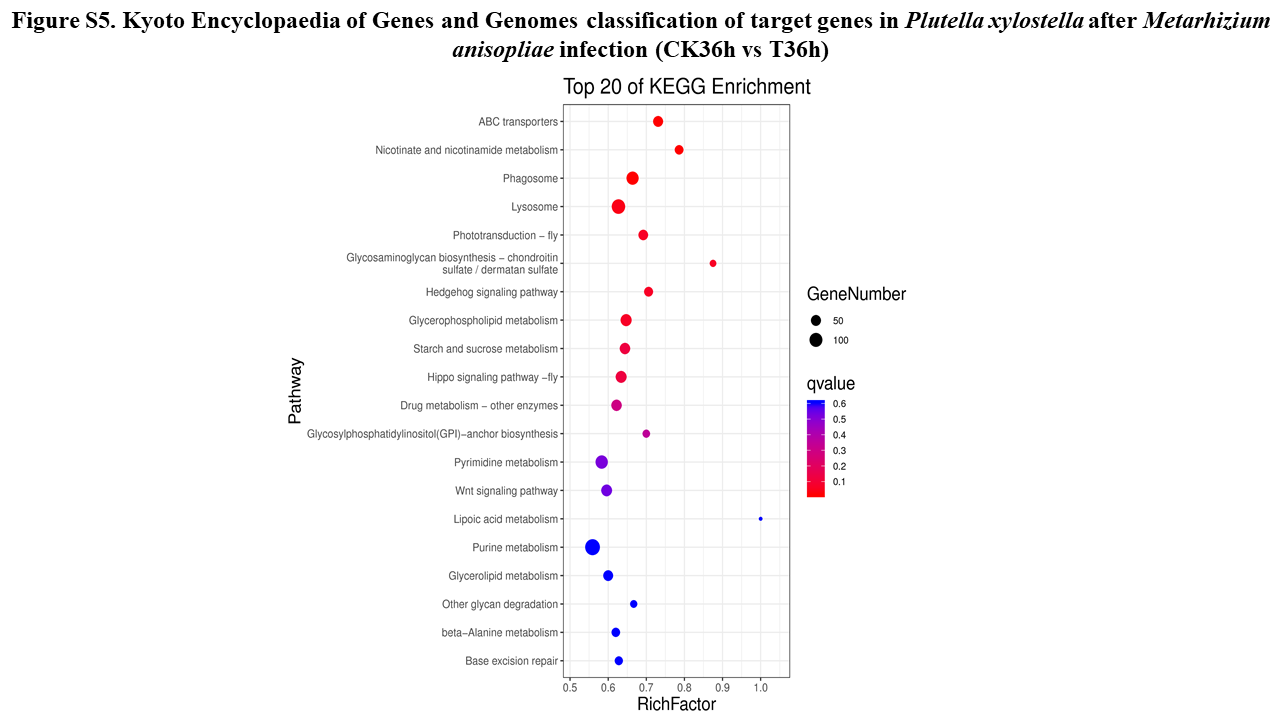

Supplement: Supplementary file 1 [file jof-07-00942-s001.zip › Figure S5 Kyoto Encyclopedia of Genes and Genomes classification of target genes in Plutella xylostella after Metarhizium anisopliae infection (CK36h vs T36h).PNG]

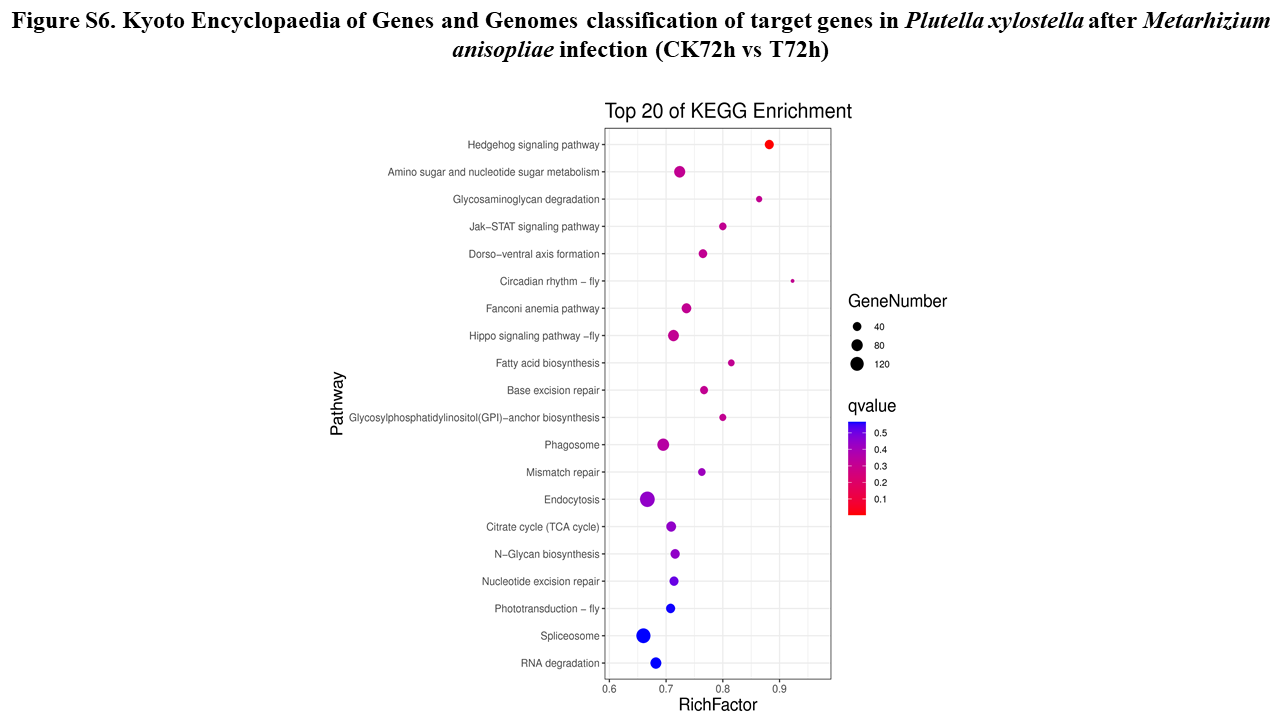

Supplement: Supplementary file 1 [file jof-07-00942-s001.zip › Figure S6 Kyoto Encyclopedia of Genes and Genomes classification of target genes in Plutella xylostella after Metarhizium anisopliae infection (CK72h vs T72h).PNG]
